# Current Challenges and Potential Strategies to Enhance Efficacy of Oral Phage Therapy in Food Animals: A Systematic Review with Quantitative Analysis

**DOI:** 10.3390/v18050544

**Published:** 2026-05-08

**Authors:** Md Ashiqur Rahman, Rebecca Abraham, David J. Hampson, Sam Abraham, Jasim M. Uddin

**Affiliations:** 1 School of Veterinary Medicine, Murdoch University, Perth, WA 6150, Australia; 35024217@student.murdoch.edu.au (M.A.R.); d.hampson@murdoch.edu.au (D.J.H.); 2 School of Medical, Molecular and Forensic Sciences, Murdoch University, Perth, WA 6150, Australia; r.abraham@murdoch.edu.au (R.A.); s.abraham@murdoch.edu.au (S.A.); 3 Centre for Biosecurity and One Health, Harry Butler Institute, Murdoch University, Perth, WA 6150, Australia; 4 Centre for Animal Production and Health, Food Future Institute, Murdoch University, Perth, WA 6150, Australia

**Keywords:** food animals, GIT, phage therapy, pH, microencapsulation

## Abstract

Phage therapy has enormous potential in combating bacterial resistance in food animals. However, its application via the oral route remains limited due to challenges associated with the gastrointestinal tract (GIT) environment and a lack of rigorous clinical trial evidence. Therefore, we systematically searched in Google Scholar, PubMed, Scopus, and Web of Science databases following PRISMA guidelines and finally identified 111 articles on oral phage therapy in food animals from where we summarized the key physiological and chemical factors of the gut environment hindering the effectiveness of oral phage therapy (OPT), examined the methods used to evaluate phage stability in the GI environment, and highlighted potential strategies to mitigate these challenges. In addition, we performed quantitative analysis to visualize in vitro pH and thermal stability patterns of phages targeting bacteria isolated from food animals and variability in buffer and incubation period across stability studies. The GIT consists of several anatomically and functionally distinct segments, where complex interactions occur among digestive enzymes, gastric acids, electrolytes, commensal microbiota, and mucosal immune components. The acidic pH of the stomach is a major barrier to successful oral phage delivery. According to our analysis of pH stability testing data from the reviewed studies, most phages targeting antimicrobial-resistant bacteria in food animals remained stable at pH 5–9 and inactivated under highly acidic (pH ≤ 2) or highly alkaline (pH ≥ 11) conditions. In addition, phages are susceptible to high temperatures (above 60 °C), digestive enzymes (e.g., pepsin, trypsin, lipases), bile salts, and host immune responses. Several in vitro laboratory techniques are available to assess phage stability under simulated GI conditions, but variations occur in the assessment protocols. Microencapsulation using alginate and chitosan has been used to protect phages from the adverse GI environment. Additionally, enteric-coated capsules, antacids, co-encapsulation with acid-neutralizing agents, consumption of alkaline water, and daily phage administration are suggested to improve phage survival and efficacy. For the successful clinical implementation of OPT in food animals, future research should focus on elucidating the molecular and physicochemical determinants of phage stability, understanding the humoral immune response to OPT, standardizing laboratory protocol for assessing phage viability, improving the scalability of encapsulation methods, and exploring other potential delivery techniques.

## 1. Introduction

Antimicrobial resistance (AMR) causes remarkable economic loss to the animal production industries and is a great concern for animal health and welfare. Globally, cumulative GDP loss due to AMR in livestock is anticipated to reach USD 575 billion by 2050 [1]. Additionally, resistant bacteria in food-producing animals pose a significant public health threat, as resistant pathogens can be transmitted to humans through direct contact or the food chain. AMR is mainly driven by the excessive and inappropriate use of antibiotics for both prophylaxis and treatment [2]. To address the crisis, phage therapy has emerged as a promising approach to treat infections caused by AMR pathogens and reduce reliance on conventional antibiotics.

Bacteriophages are ubiquitous viruses that attach to bacterial cell-surface receptors, inject their genetic material, undergo intracellular replication, and ultimately lyse bacterial cells or establish long-term lysogeny [3]. They can kill both antimicrobial-susceptible and resistant bacteria, with minimal or no harmful effects on the microbiota in animals. Their self-replication ability within the bacterial host enables therapeutic efficacy with single-dose administration. Furthermore, phages possess immunomodulatory properties and can be formulated into synergistic, broad-spectrum therapeutic cocktails, either in combination with other phages or conventional antibiotics [4]. Despite their considerable therapeutic potential, bacteriophage applications remain mainly confined to experimental studies and compassionate use, primarily due to inconsistent efficacy in clinical settings [5,6]. The inconsistent efficacy in clinical settings is primarily due to limited pharmacokinetic understanding, adverse immune reactions in response to phage administration, and complex interaction with resident microbiota [7]. Other factors that hinder its widespread clinical application include instability across delivery routes, reduced phage concentration at the target site due to degradation, dilution, or clearance, the development of phage-resistant mutants driven by strong selective pressure on target bacteria, narrow host specificity as both precise pathogen identification and individualized phage preparation required, time consuming production systems, a lack of production standards, and the absence of relevant regulatory guidelines [8,9]. Among the available administration routes, oral delivery stands out as the most practical, convenient, non-invasive, and scalable approach for treating food animals [10,11]. Although phage research has expanded significantly in recent years, only a limited number of studies have explored determinants of phage efficacy in delivery routes, strategies to enhance phage delivery and bioavailability in food-producing animals.

In light of this, the systematic review aims to explore the intricate environment of the GIT in terrestrial food-producing animals (pigs, cattle, and poultry), key factors influencing the viability of OPT, assessing conventional methodologies for evaluating phage stability within the gut environment, and delineating potential strategies to optimize the efficacy of OPT. The synthesized information will provide valuable insights for researchers, supporting the development of sustainable and convenient oral phage-based therapeutics in animal health.

## 2. Literature Search and Analysis

**Figure 1:**
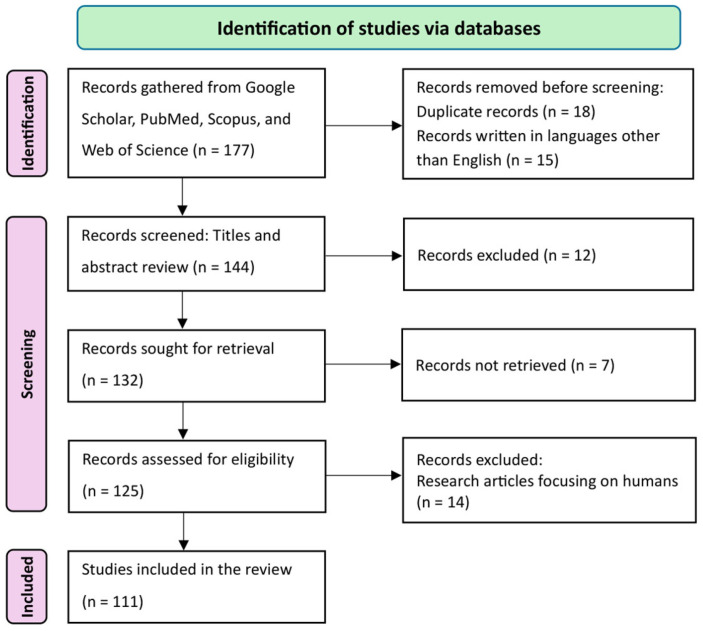
PRISMA flow diagram of the review process for screening and selecting relevant publications on oral bacteriophage therapy to control AMR in food animals.

The systematic review, conducted without meta-analysis, was performed in accordance with the PRISMA guidelines [12]. A primary literature search for published research till December 2025 was performed by the first author on Google Scholar, PubMed, Scopus, and Web of Science databases to find studies on the oral application of bacteriophage therapy in food animals, including pigs, cattle, ducks, and chickens. All selected databases were accessed by using Boolean keyword search terms. The key search terms included: “antimicrobial resistance or AMR in food animals or livestock or farm animals or swine or pigs or cattle or poultry,” “Gastrointestinal tract or GIT or digestive tract environment or intestinal condition or physiology in food animals or livestock or farm animals or swine or pigs or cattle or poultry”, “pH or temperature or enzymes or ions or immune status or microbes or bacteria in food animals or livestock or farm animals or ruminants or non-ruminants or swine or pigs or cattle or poultry, determinants or factors of bacteriophage or phage viability or stability or survival or persistence in gastrointestinal tract or GIT or digestive tract in food animals or livestock or farm animals or ruminants or non-ruminants or swine or pigs or cattle or poultry”, “effect or outcome of pH or temperature or bile salts or enzyme or ions of phage viability or stability or survival or persistence in food animals or livestock or farm animals or ruminants or non-ruminants or swine or cattle or poultry”, “pH or thermal or ions or enzymatic or storage stability or survival or persistence of bacteriophage or phage test or technique or assay or methods or assessment or evaluation of food animals or swine or pigs or cattle or poultry”, and “strategies or techniques or methods or assay for enhancing or improvement phage stability and efficacy in in gastrointestinal tract or GIT or digestive tract in food animals or swine or pigs or cattle or poultry”. Original research articles, review papers, and non-peer-reviewed documents (theses and dissertations) were included in the initial screening. Non-peer-reviewed documents were initially screened to ensure more comprehensive coverage of the available evidence, as some studies remain unpublished in peer-reviewed journals. A total of 177 articles were retrieved. Firstly, duplicates and articles not written in English were removed. The titles and abstracts were then primarily reviewed to select relevant articles. Subsequently, relevant articles that could not be retrieved due to a lack of full-text availability were removed. Finally, research involving humans was removed. The remaining articles (*n* = 111) underwent a comprehensive review to extract pertinent information, including experimental species, phage name, host bacteria, host strain, buffer used, incubation period, incubation temperature, pH studied, pH stable, temperature studied, phage temperature stability, titer reduction due to pH and temperature variability, and microencapsulation status, which was systematically organized and incorporated into the manuscript. The phage pH and thermal stability data were obtained from recent studies published till 2025 and analyzed in R statistical software version 4.5.1. The ggplot2 and pheatmap packages were used to create the heat map, box plot, and bar charts. Other figures were created using Microsoft PowerPoint software. The study flow diagram is presented in Figure 1.

## 3. The Gastrointestinal Environment

**Figure 2:**
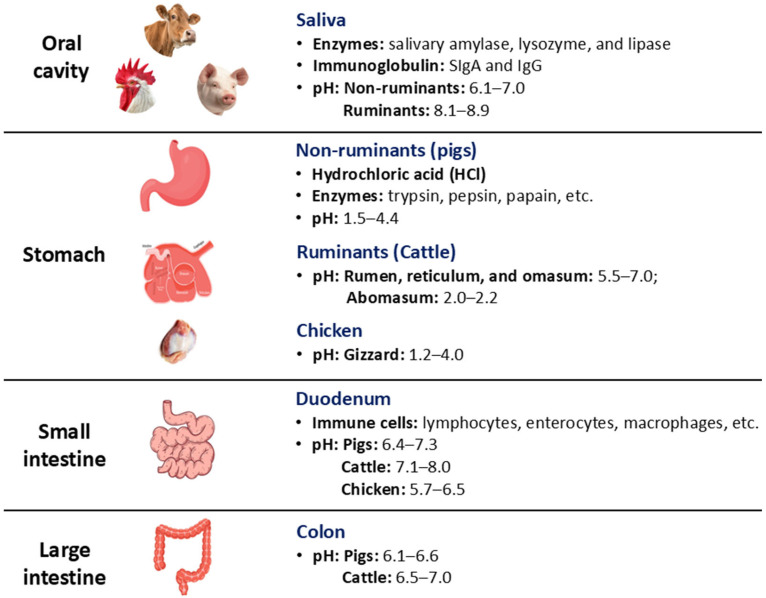
Diagram summarizing the gastric and intestinal environment of food animals.

The GIT of food animals forms an intricate ecosystem that continues from the oral cavity to the anus and is crucial for digestion, nutrient absorption, and excretion. In GIT, tissues, dietary components, enzymes, and microorganisms interact with each other and function to support activities. In the oral cavity, saliva, a distinctive fluid that comprises primarily enzymes consisting of salivary amylase, lysozyme, and lipase, is secreted from the salivary glands, which aid in lubrication, digestion of food, stabilization of acid-sensitive drugs, manage supersaturation of calcium and phosphate concentrations, and prevent microbial growth in the oral cavity [13,14]. Saliva also contains immunoglobulin (SIgA and IgG) molecules that degrade microbes, including viruses, bacteria, fungi, and parasites [15]. The pH of saliva differs among species, ranging from 8.1–8.9 in ruminants and 6.1–7.0 in non-ruminants [16,17]. The principal function of the stomach is to break down feed particles and facilitate digestion. The ruminant and non-ruminant stomachs are substantially different in many aspects, such as structure, physiology, and microbiology. The stomach of monogastric animals, such as pigs, secretes hydrochloric acid (HCl) and proteases, including trypsin, pepsin, and papain, creating an acidic environment with a pH range of 1.5–4.4, which helps digest food and may degrade acid-sensitive drugs [18]. This acidic pH also denatures proteins and aids microbial control within the gastric environment [19]. The gizzard, which is considered the stomach of chickens, has a pH range from 1.2 to 4.0 [20]. In contrast, the ruminant stomach has four compartments where the pH in three of the compartments is close to neutral (5.5–7.0), which helps the microbes to digest fiber. The pH is acidic (2.0–2.2) only in the abomasum (the fourth compartment) and aids in acidic and enzymatic digestion (Figure 2). GIT is colonized with a diverse population of microbes, including bacteria, viruses, phages, protozoa, fungi, and archaea, and varies in non-ruminants and ruminants [21]. The stomachs of non-ruminants harbor bacteria belonging to the genera *Lactobacillus*, *Enterobacteriaceae*, *Enterococcus*, *Propionibacterium*, *Bacteroides*, *Bifidobacterium*, *Clostridium*, and *Staphylococcus* [22]. On the other hand, the diverse array of bacteria present in the ruminant stomach belongs to the genera *Clostridium*, *Selenomonas*, *Eubacterium*, *Butyvibrio*, *Ruminococcus*, *Anaerovibrio*, *Megasphaera*, *Bacillus*, *Streptococcus*, *Prevotella*, *Escherichia*, *Salmonella*, *Ruminobacter*, and *Succinivibrio*. Fungi found in the rumen belong to the family *Neocallimastigomycota*, and protozoa belong to the families *Isotrichidae* and *Ophryoscolecidae* [23]. The principal role of microbes is to facilitate the digestion of ingested substances by producing enzymes. In addition, they regulate key functions related to metabolism, immunity, and other physiological functions. Moreover, the microbes act as a reservoir of certain genes, which they transfer to other microbes through horizontal gene transfer [24]. Bacteriophages or phages found in the distal part of the GIT lyse the overgrown bacteria to maintain homeostasis of the overall bacterial population, and provide immunity to animals against superinfection by lysogenization of bacterial cells [25].

The small intestine is the primary site of chemical digestion and drug absorption. Orally applied drugs dissolve by mixing with bile and pancreatic secretions and subsequently transfer the molecules from the gut lumen across the gut wall to the body tissues. The pH inside the small intestine varies between food animals, ranging from around 6 to 8, including pigs (6.4–7.3), chickens (5.7–6.5; duodenum), and cattle (7.1–8.0) [20,26,27]. Moreover, the neutral pH, increased surface area, and long resident time (2–3 h) help in nutrient and drug dissolution and absorption. Likewise, the other GI parts, including the small intestine, contain microbes and various immune cells, such as lymphocytes, enterocytes, macrophages, and other cells that protect the surface epithelium, allowing it to digest and absorb feedstuffs by defending it from infection. The undigested feed particles are fermented in the large intestine by the commensal microflora of the colon [16]. The pH of the colon (cattle: 6.5–7; pigs: 6.1–6.6) is comparatively lower than the lower parts of the small intestine, which might be due to the presence of bacteria and their fermentation products (cellulose and proteins into volatile fatty acids) [26,27]. Additionally, the immune system produces IgA antibodies and large numbers of regulatory T cells, which recognize the commensal microflora without expelling them [28].

Several components with diverse functions are present in the GIT lumen of food animals. The interaction between orally administered phages and the lumen contents are crucial for the phage stability and efficacy. Before selection and application of phages orally, analyzing their stability in gut conditions is warranted. Thus, the next sections focus on the components that affect phages from a therapeutic point of view, techniques for measuring stability, and ways to enhance their efficacy when administered to food animals.

## 4. Determinants of Phage Stability in the GIT

Phages consist of nucleic acid and structural and non-structural proteins. The survivability of orally applied phage cocktails in the GIT is influenced by multiple factors, including pH, temperature, ions, enzymes, bile salts, and other environmental conditions.

### 4.1. pH

The pH is the most critical determinant of phage stability in the GIT of food animals [29]. It varies widely across different GI compartments in both ruminant and non-ruminant species, with the stomach being a major barrier during oral phage administration. Highly acidic conditions of the stomach can denature phage surface proteins [30], which impair their ability to reach and lyse target bacteria [31]. In vivo studies in food animals such as pigs, cattle, and chickens have reported that oral administration of liquid, unprotected phage formulations in animals lead to reduced phage viability [11,32,33]. The acid stability of phages is typically evaluated in vitro prior to in vivo applications.

**Figure 3:**
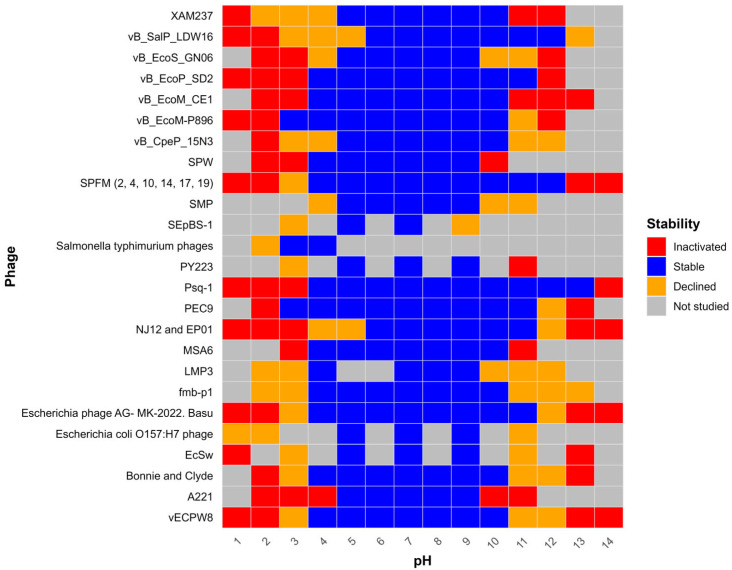
In vitro pH stability of lytic phages against bacterial pathogens in food animals (data collected from research articles have been incorporated as Supplementary File).

A heatmap was constructed to visualize in vitro pH stability patterns of phages targeting bacterial pathogens isolated from pigs, chickens, cattle, and ducks (Figure 3). Phage stability was categorized into four groups: stable, declined, inactivated, and not studied, and represented using discrete color coding.

Most studies evaluated phage viability across a broad pH range, from highly acidic (pH 1) to alkaline conditions (pH 14). Only Wanasawaeng et al. [34] and Albino et al. [35] specifically examined stability at low pH. In several studies, odd-numbered pH values (1, 3, 5, 7, 9, 11, and 13) were used to assess the stability of phages such as PY223, EcSw, and *E. coli* O157:H7 phage [36,37,38].

*E. coli* (*n* = 13) and *Salmonella* spp. (*n* = 6), the two most important bacterial pathogens causing foodborne illness and contributing to AMR transmission, were the most frequently targeted pathogens (Figure 3). Phage stability was also evaluated against porcine meningitis-causing *Streptococcus suis* (*n* = 2) and cattle mastitis-causing *Staphylococcus aureus* (*n* = 2). Additionally, *Bordetella bronchiseptica*, *Listeria monocytogenes*, and *Clostridium perfringens* were each represented in a single study.

Based on the included studies in the heatmap, phages targeting bacterial pathogens in food animals exhibited stability within a pH range of approximately 5–9. In contrast, inactivation or reduced stability was more frequently observed under a highly acidic pH (1–2) or a highly alkaline pH (≥11). However, the included studies demonstrate substantial heterogeneity across phage species, bacterial hosts, animal models, buffer systems, pH exposure durations, temperature conditions, and titration methodologies (Supplementary Table S1). Such variability may influence reported stability profiles and limit direct comparability across studies. At pH 3, only vB_EcoM-P896, *S.* Typhimurium pool phages, and PEC9 remained viable [35,39,40]. On the contrary, vB_SalP_LDW16, SPFM (2, 4, 10, 14, 17, and 19), Psq-1 were only stable at pH 12 [41,42,43].

Phage viability reduction was expressed either as a log or a percentage. At pH 2, LMP3 and XAM237 phages lose 50% and 70% of their viability [30,44]. After 1 h of incubation at the same pH, an 8.5 log reduction in fmb-p1 phage was documented [45]. Only A221 was inactive at pH 4 [46].

In addition, certain research explored phage stability in simulated gastric fluid (SGF). For instance, Zhang et al. [47] revealed that a *S.* Enteritidis phage lost activity within 20 and 30 min in SGF (3.2 mg/mL pepsin in 0.2% NaCl) at pH 2.0 and 3.0, respectively. Similarly, Yin et al. [48] also noted complete inactivation of the enterohemorrhagic *E. coli* O157:H7 phage PNJ1901 after 15–30 min of incubation in SGF at pH 2.0–2.4. The reduction in phage survival might be due to the denaturation of phage outer proteins caused by the extreme acidity of SGF [49]. In contrast, Chanthavong et al. [50] tested a phage cocktail against multi-drug-resistant *E. coli* strains (resistant to amoxicillin, oxytetracycline, neomycin, sulfamethoxazole-trimethoprim, gentamicin, cephalexin, enrofloxacin, and colistin) responsible for diarrhea in piglets and observed no significant activity loss when exposed to SGF at pH 2.0.

Phage stability at highly acidic pH levels is highly variable. Therefore, both in vitro testing of phage candidates’ survival at lower pH levels and the development of protection strategies should be implemented to ensure that phages pass through the stomach lumen and are released at the intestinal target site.

### 4.2. Temperature

Temperature profoundly impacts bacteriophage structural integrity, phage latency, and infection efficiency. Therefore, thermal stability is considered a crucial parameter for phage storage and cocktail formulation.

**Figure 4:**
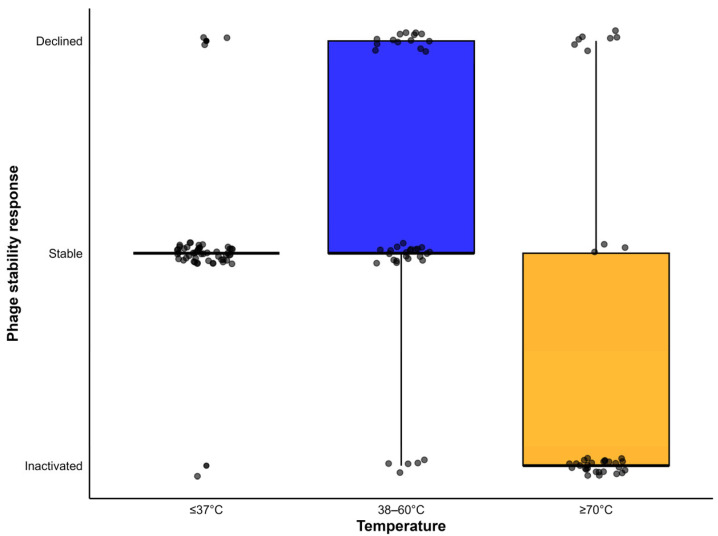
Thermal stability of lytic phages targeting a range of bacterial pathogens in food animals (Data collected from research articles have been incorporated as Supplementary File).

A box plot was constructed to summarize the thermal stability of phages targeting food animal bacterial pathogens across three temperature groups (≤37 °C (2–37 °C), 38–60 °C, and ≥70 °C (70–100 °C)) (Figure 4). Overall, most phages targeting bacterial isolates from food animals remain stable within the range of 4–60 °C. The box plot indicates that phages generally exhibited greater stability at lower temperature ranges (≤37 °C) (Figure 4). Taj et al. [51] also reported that T4 phages exhibit optimal bacteriolytic activity at 37 °C. As the gut temperature maintains around 37 °C, phages tolerate and easily pass through the gut lumen of food animals.

However, phage stability during storage is affected by temperature. The titer of an *E. coli* O157:H7 phage declined when stored at −20 °C and −80 °C for 90 days [38]. Hatch and Warren [52] revealed that phages should not be stored at −20 °C because ice crystals form at this temperature, which can lyse phages. Olson et al. [53] recommended that phage should be stored at 4 °C for short-term (up to four weeks) and at −80 °C for long-term preservation. Storage at ultra-low temperature (−80 °C) minimizes biochemical degradation, limits microbial contamination, and prevents loss of infectivity [54]. However, phage stability under frozen storage conditions varies depending on phage morphology (tailed phages are more stable), phage family, the use of cryoprotectant, and repeated freeze–thaw cycles [55]. Reviews of the literature highlighted that most phages were relatively stable at 38–60 °C (Figure 4).

Significant variation of phage stability was exhibited at 60 °C. Both complete stability and titer reduction were observed at the temperature. For example, phage titer decreased by 1.5–4 log_10_ after an hour of incubation at 60 °C [40,56,57,58]. Similarly, Zhang et al. [39] and Easwaran et al. [37] reported approximately 20% and 40% reductions in phage titer, respectively, after one hour of incubation at 60 °C.

On the other hand, inactivation of most phages was observed at ≥70 °C (Figure 4). This might be due to the denaturation of capsid proteins and the degradation of nucleic acids [59]. Notably, SPFM10, a bacteriophage that can lyse *Salmonella* spp., is the only phage reported to be stable up to 80 °C [42]. The findings indicate that the thermal tolerance of phages targeting bacteria in food animals varies between studies. Thus, prior assessment is essential for standard phage formulation and storage.

### 4.3. Ions

The stomach and small intestines in food animals secrete various ions, including bicarbonate, chloride, sodium, and potassium, which are essential for digestion and nutrient absorption [21]. The ions may not have a direct effect on phage stability, but their release in the small intestine neutralizes acid and enhances pancreatic enzyme activity [60]. Despite their physiological importance, a few studies have explored the impact of ionic conditions on phage stability. Early research primarily focused on sodium chloride (NaCl) due to its potential interference with phage absorption in bacteria [61]. These studies reported that extreme NaCl concentrations can significantly reduce phage viability. For instance, Karami et al. [58] reported a 0.73 and 0.88 log_10_ PFU/mL reduction in *E. coli* phage AG-MK-2022 Basu titer at NaCl concentrations of 11% and 13%, respectively. However, no reduction was observed at lower NaCl concentrations (≤9%). On the other hand, Abdelaziz et al. [62] reported that incubating phages for 24 h at 3.5% salt concentration reduced phage viability more than incubation at 0.1% salt concentration. Analogously, Wanasawaeng et al. [34] observed a significant decrease in the titer of *Salmonella* targeting phage SEpBS-1 in highly concentrated (5%) NaCl solutions compared to lower concentrations (2%). Therefore, phage ionic tolerance should also be measured before selecting for oral administration.

### 4.4. Enzymes and Bile Salts

Phages are generally resistant to digestive enzymes [32]; however, some are sensitive to proteolytic enzymes in the stomach and small intestine [63]. Gastric pepsin, along with HCl, damages peptide bonds and degrades surface proteins, compromising phage stability [63]. A study by Imklin et al. [2] assessed the stability of five AMR *E. coli* phages in SGF containing pepsin (3.2 mg/mL), found that three phages were completely inactivated. Phage PNJ1901 (Host: *E. coli* O157:H7) introduced to SGF (pepsin: 3.2 mg/mL) was also mostly inactivated after 15 and 30 min of incubation [48]. Bacterial lipases may also degrade phage envelopes in the intestine [64]. The pancreas secretes trypsinogen into the duodenum; brush border enterokinase activates it to trypsin, degrading surface proteins of certain filamentous phages such as coliphage P1 [65]. Conversely, a few phages, including T4, T2, UZ1, lambda, and a *Vibrio cholerae* phage, remain resistant to trypsin [32]. Accordingly, phages resistant to trypsin could be sensitive to other proteases, including proteinase K or papain [66].

Bile, produced by hepatocytes and stored in the gallbladder, is secreted into the small intestine for fat solubilization and contains bile salts like sodium cholate and sodium deoxycholate. Orally administered phages that withstand gastric acidity subsequently enter the duodenum and are exposed to high concentrations of bile salts. Bile salts can lower phage contact rates with their host bacteria, viability, and adsorption [67]. Yin et al. [48] observed a time-dependent decline in Shiga toxin-producing Enterohemorrhagic *E. coli* O157:H7 phage PNJ1901 (isolated from chicken faeces) titer following bile salt exposure. Similarly, Scanlan et al. [67] reported that the viability of phage PP01 (Host: *E. coli* NCTC12900) decreased to 40% after 24 h of exposure to bile salts. Nonetheless, most phages, particularly those infecting *Enterobacteriaceae*, exhibit minimal or moderate sensitivity to bile components, likely due to their lack of lipid membranes [32,48]. Therefore, phage stability testing should not be limited to only pH and temperature when aimed at oral application; ions and bile salt stability testing are also equally important for measuring their suitability.

### 4.5. Other Determinants

The intestinal mucosa contains several immune cells that secrete specific antibodies to maintain intestinal homeostasis. Specifically, if orally administered phages cross the intestinal mucosa and enter the lamina propria, the gut produces specific immunoglobulin A (IgA) that neutralizes the phages [68]. Besides, phages that enter the bloodstream from the lamina propria can interact with immune cells and induce innate and adoptive immune responses [69]. Phages activate both pro-inflammatory (up-regulation of IL-1α, IL-1β, and TNF-α) and anti-inflammatory (up-regulation of Interleukin-1 Receptor Antagonist and Suppressor of Cytokine Signaling 3) immune responses in animals [70]. Therefore, elevated serum IgG levels following phage administration further indicate systemic humoral immune activation [71]. Besides, Majewska et al. [68] reported that oral T4 phage administration in mice induced gut-derived IgA, leading to complete lysis of active phages. Although several studies reported phage application induces humoral immune response, the exact mechanism of the response and effect on treatment efficacy is poorly understood [72].

Endogenous phages can significantly influence the efficacy of oral phage therapy by interfering with the ability of therapeutic phages to bind to their target bacteria. Additionally, they can exert selective pressure on bacterial surface receptors, leading to downregulation or modification of the receptors, which lowers phage susceptibility and therapeutic efficacy [73]. Endogenous phages and the resident bacterial community create ecological constraints that can limit the adsorption, replication, and lytic activity of externally administered phages (Muneeb et al., 2025) [74]. Moreover, endogenous phages may harbor AMR genes or virulence factors that can be spread within the microbiome during interactions between endogenous and therapeutic phages [8]. If a single phage is administered repeatedly, target bacteria also become resistant through several mechanisms, including absorption inhibition, prevention of irreversible takeover of host metabolism, activation of abortive infections, and CRISPR (Clustered Regularly Interspaced Short Palindromic Repeats) mediated immunity [75]. Studies on endogenous phages in livestock remain limited; however, emerging evidence suggests they play beneficial roles within the host gut ecosystem [76]. Phage dosage is a critical determinant of therapeutic success in oral phage delivery. Studies in poultry have demonstrated that relatively higher phage doses that are sufficient to maintain an effective multiplicity of infection (MOI) can significantly reduce target bacterial populations in the GIT. According to the findings of Atterbury et al. [77], *S*. Enteritidis and *S*. Typhimurium challenged birds treated with phages at 10^9^ PFU/bird did not show a substantial reduction of bacterial load. In contrast, treatment with 10^11^ PFU/bird led to a substantial reduction of both *Salmonella* species in the cecal contents [78].

## 5. Evaluation Techniques for Phage Stability

Evaluating phage stability within the GIT of food animals is essential for effective oral phage therapy. Prior studies have investigated phage stability under various conditions, including pH, temperature, bile salts, SGF, and NaCl concentration [2,35,46,58]. However, most research has focused on pH and thermal stability, with limited exploration of phage tolerance to digestive enzymes, ionic conditions, and host immune responses [34,37,72,79]. Importantly, methodologies used across studies vary considerably. General approaches and methodological differences have been discussed in this section. Phage pH tolerance is assessed by adjusting buffer solutions to defined pH values using 1 M NaOH or HCl. The ionic solutions are able to dissociate fully and change pH quickly; therefore, the solutions are mostly used to adjust pH in phage efficacy experiments [80]. Buffer solutions for pH stability assessments, including SM (Saline magnesium) buffer, LB (Luria-Bertani) broth, PBS (Phosphate buffer saline), TSB (tryptic soy broth), TM (Tris-Magnesium) buffer (50 mM Tris, 10 mM MgSO_4_), NB (Nutrient broth), THB (Todd–Hewitt broth), and physiological saline, were utilized previously for pH stability assessments [34,40,44,62,81,82,83].

**Figure 5:**
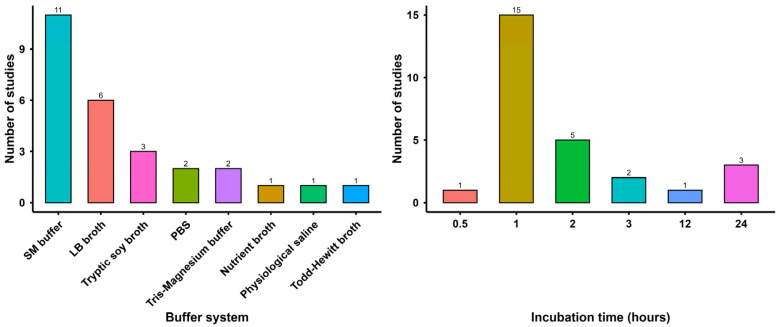
Frequency distribution of buffer and incubation times used in phage pH stability studies (*n* = 27) targeting food animal bacterial pathogens.

However, the SM buffer was the most frequently applied buffer solution across studies (Figure 5) because it offers a more optimal ionic composition (presence of magnesium and sodium ions) and near neutral pH conditions for maintaining phage infectivity by stabilizing protein structures than other buffer solutions [41,42,46,56,58,84,85,86]. Moreover, the SM buffer contains gelatin, which stabilizes phage particles during preservation [87]. As phages are sensitive to low pH levels, Albino and his colleagues assessed stability to low acidic pH ranges (pH 2.0–4.0) [35]. After pH adjustment, high-titer purified phage suspensions are mixed with buffers in separate tubes and incubated at 37 °C for different durations ranging from 30 min to 24 h [34,38]. Although incubation times vary, 1 h is commonly used in most studies (Figure 5), as stomach residence time in monogastric animals is relatively short [88]. Incubation period also varied with room temperature (25 °C), and 41 °C in some studies [34,39,42]. Viability is determined by the double-layer agar (DLA) method, a common method used for bacteriophage enumeration, and titer reduction is calculated based on pre- and post-incubation concentrations [40,56]. Buffer has a major impact on the phage infectivity [84], but most of the studies comparing pH stability of phage without considering buffer variability. Therefore, studies need to be designed to evaluate buffer-dependent phage titer variability when exposed to several pH levels.

Stability against gastric enzymes is commonly assessed by incubating purified phage preparations with SGF, formulated by combining 3.2 mg/mL pepsin with 0.2% (*w*/*v*) NaCl at pH 2.5. SGF is used mainly for reflecting the physiological state of the intestine, which contains necessary gastric enzymes. Pepsin is the principal component of SGF as the gastric enzyme involved with crucial functions such as protein, peptide, and antibody digestion. Moreover, the acidic pH (2.5) of SGF ensures optimal peptic activity [89]. Phage suspensions are added to pre-warmed SGF (37 °C) and incubated for a few minutes to several hours, where shaking during incubation is recommended [48]. Variation in incubation time is also observed in SGF for pH stability testing. Following incubation, phage viability is quantified using the DLA method [2,48,90]. However, prolonged exposure to pH decreases viability, necessitating standardized incubation [91].

The thermal stability of bacteriophages is determined by incubating phage suspensions at various temperatures for different durations ranging from 10 min to 90 days, followed by titration using the DLA method [79,81]. Earlier studies have incubated purified phage preparations at temperatures ranging from 4 °C to 100 °C, while maintaining a pH of 7.5 [42,82]. The studied temperatures were selected considering the phage structure, application, and storage conditions [92]. Experiments were typically conducted in triplicate to calculate the mean phage titer [43,57]. Wide variation was observed in the temperature levels studied, which results in interpretation ambiguity and declined reproducibility. Therefore, standard protocol development for thermal stability assessment is essential.

The effect of salinity on phage stability is rarely determined, which is measured by incubating purified phage with NaCl solutions of varying concentrations (ranging from 0.1% to 11%) at 37 °C for up to 24 h [34,45,58,62]. A previous study reported that NaCl concentration greater than 1% can inactivate some phages, and marked tolerance and viability variations are observed among phages [93]. Sterile distilled water is used as the negative control [34] and the residual phage titer is determined using the DLA assay [62].

Furthermore, phages are exposed to the feed particles, several enzymes, immune cells, and other digestive contents together upon oral administration. Their combined interaction may change the studied parameters, phage state, and viability. Therefore, besides testing single-factor or digestive components stability testing, combined multifactorial stability testing could help to select highly stable and target-site-releasing candidate phages.

Few studies using animal models are employed to assess the host immune response to phage therapy. After oral phage administration, serum and intestinal mucosa samples are collected to assess humoral and cell-mediated immune responses by Enzyme-linked immunosorbent assay (ELISA) and immunohistochemistry [72,94]. Particularly, inflammatory factors IL-1β (interleukin-1β), IL-4, IL-10, IL-17, TNF-α (tumor necrosis factor- α), IFN-γ (Interferon-γ), TGF-β (transforming growth factor β), Toll-like receptor 4 (TLR-4), TLR-9, and serum immunoglobulins (IgA, IgG, IgM) were measured in a study conducted in a porcine model [71]. The rise of IFN-γ, IL-1β, IL-17, TGF-β, IgA, IgG, and IgM, drop of IL-4 and IL-10, and activation of TLR-4 and TLR-9 levels both in serum and intestinal mucosa indicate host immune response against phage [71]. TGF-β is a cytokine that is crucial for maintaining immune homeostasis. TLRs detect degraded nucleic acids, denoting inflammatory factor production. Moreover, an increase in serum IgA, IgG, and IgM indicates transfer of phages from the intestinal cavity to the circulatory system [71]. However, another study demonstrated that the phage application in an infected mammary gland reduced cytokines IL-6 and TNF-α, indicating effective alleviation of the inflammatory response [95]. Few studies also harvested spleens for analyzing innate immune cell (NK cells, eosinophils, neutrophils, macrophages, and dendritic cells) populations, using flow cytometry to evaluate both cell frequencies and absolute counts across different innate subsets [72,94]. Only a few studies have explored the recipient (food animals) immune response to phage therapy, which results in a limited understanding of the actual effect. Although phage immune response studies in porcine, chicken or cattle models are not always feasible, more studies are needed to enhance understanding and acceptance for mass application.

## 6. Strategies for Enhancing Phage Cocktail Stability and Efficacy

The oral route is the most common and convenient means of delivering therapeutics to food animals to control acute GI infections. However, the bioavailability of orally administered bacteriophages is often limited due to various gastrointestinal factors discussed earlier. For enhancing the bioavailability of phage cocktails in the oral route and getting maximum output of phage therapy, several strategies such as microencapsulation, enteric capsulation, gastric acid neutralizers, probiotics, etc., are used.

### 6.1. Encapsulation

Microencapsulation is a widely applied strategy for protecting phages from gastric degradation, particularly from gastric acid, enzymes, and microflora [48]. It enables targeted release, prolongs shelf life, and enhances phage stability during storage [63,96]. Several biopolymers, including cellulose, alginate, chitosan, carrageenan, gelatin, pectin, and starches, have been used for encapsulation [97]. Carrageenan has been extensively used in the food industry but may exhibit unwanted immunological responses and adverse effects on living cells, therefore not suitable for animal use [98]. On the contrary, cellulose, gelatin, and starch do not dissolve in the gut because of the absence of secretion of necessary enzymes [98]. Pectin is resistant to low pH, gastric enzymes, and intestinal microflora, but it becomes swollen in liquid media, which results in low drug release into the GI lumen [99]. Among these, alginate is frequently used for encapsulating phages targeting GI pathogens [46,48,85]. Alginate can be derived from marine sources, a non-toxic, linear copolymer of β-D-mannuronate and α-L-guluronate, known for its ability to neutralize acids and sustain phage viability in the gut [100,101]. Due to alginate instability in the presence of cations like sodium and magnesium, chitosan is commonly used as a final coating [90]. Chitosan is soluble in acidic media and exhibits better biocompatibility [101].

**Figure 6:**
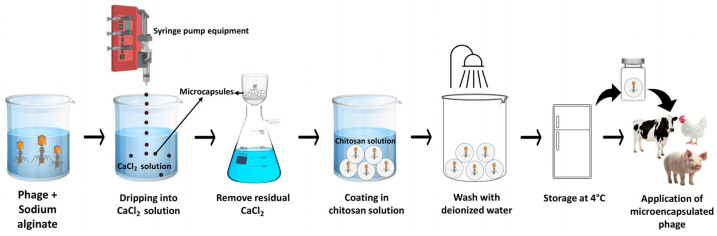
Schematic representation of the microencapsulation process and its therapeutic applications in food animals.

The encapsulation process typically involves suspending phages in sodium alginate solution, which is then added dropwise into a CaCl_2_ solution at a uniform rate to form microcapsules [101]. CaCl_2_ acts as a cross-linking agent, used to connect independent linear molecules to form microcapsules [48]. Afterwards, the microcapsules are washed and filtered to remove residual CaCl_2_ [48]. Finally, microcapsules are placed in a chitosan solution for several minutes (typically 20 min), washed with deionized water, and stored in the refrigerator (4 °C) for therapeutic use (Figure 6) [85].

During encapsulation, several factors need to be considered carefully. Firstly, the concentration of sodium alginate and CaCl_2_ should be adequate. A low sodium alginate concentration cannot sufficiently react with CaCl_2_, resulting in low encapsulation efficiency [48]. Moreover, a high concentration of sodium alginate forms a viscous solution that cannot pass properly through the pinhole of the syringe, leading to atypically shaped microcapsules [48]. Besides, low concentrations of CaCl_2_ cannot cross-link properly, and high concentrations increase the hardness of microcapsules [48]. Therefore, proper optimization of the encapsulation conditions is essential.

Recent animal studies suggest that microencapsulation may be an effective technique for the success of phage therapy in GI infections. Pigs diagnosed with *S.* Typhimurium, treated with a microencapsulated anti-*Salmonella* phage cocktail (∼10^9^ PFU/mL), significantly reduced bacterial concentrations in the cecum (1.4 log_10_ CFU/mL; Control: 2.9 log_10_ CFU/mL) and ileum (1.0 log_10_ CFU/mL; Control: 2.7 log_10_ CFU/mL) [102]. Similar results were reported by Saez et al. [103], who found that oral administration of microencapsulated phages via feed and gavage significantly decreased *Salmonella* spp. colonization in the ileal and cecal contents compared to the control group. Moreover, oral administration of microencapsulated phages to weaned piglets infected with *E. coli* improved daily weight gain and alleviated diarrheal symptoms [46,85]. Similarly, encapsulated phage effectively controlled diarrhea, resulting in no weight loss in the phage-treated experimental animals (rats) [104].

It has been found that chitosan-alginate encapsulated phages remained significantly stable (*p* < 0.01) under simulated intestinal conditions (37 °C, pH 2) than non-encapsulated phages [101]. A study comparing the therapeutic efficacy of orally administered sodium alginate/calcium carbonate (CaCO_3_)-coated phages with free phages to control *Salmonella* spp. infection in broiler chickens found that microencapsulation significantly improved phage survival in the GIT [100]. Encapsulation also prolonged phage retention (15 min to 2 h) in the cecum, enhancing therapeutic efficacy [48,100]. On the other hand, it has been reported that chitosan-alginate encapsulation reduces the titer of 0.9–1.3 log_10_ PFU/mL [101,105]. To overcome the obstacle, substances such as k-carrageenan, honey, or gelatin have been used additionally to increase matrix viscosity and improve phage stability [90,96]. However, more studies need to be conducted on food animals, determining the release rate of the encapsulated phages from the microparticles in the gut, the variability of the extent of acid protection and the overall efficacy of the suggested technique. Moreover, encapsulation is a time-consuming and complex technique which requires a skilled person and advanced laboratory apparatus. Further research needs to focus on improving the encapsulation technique so that less effort, skill, and laboratory equipment are required to perform the technique.

### 6.2. Other Suggested Techniques to Overcome the GIT-Associated Limitations

Several additional strategies have been employed to enhance phage passage through the GIT lumen. Acid neutralizers, such as CaCO_3_, help maintain phage viability in the stomach. Multiple animal studies have administered CaCO_3_ prior to phage administration to reduce gastric acidity, supporting phage survival and transit to the intestinal lumen [77,90,100,106,107]. Carbonate ion dissociates from CaCO_3_ and diffuses, which increases the pH as a role of an antacid, thus protecting the bacteriophages [100]. Not only an acidic environment or HCl, but also enzymes often denature and digest proteins, including phages, so using only an acid neutralizer cannot protect phages from an adverse GI environment. Therefore, CaCO_3_ could be co-encapsulated in alginate-based systems to improve acid resistance. Colom et al. [100] and Ma et al. [90] showed that CaCO_3_ incorporation into chitosan-coated alginate microspheres enhanced phage stability under acidic conditions. However, comprehensive research is needed on phage viability, gastric retention time, and release rate due to CaCO_3_ inclusion with encapsulated phage formulation.

Additionally, providing slightly alkaline drinking water to animals makes a favorable gut passage for phages [32]. Nevertheless, several studies recommend administering multiple doses of phage instead of a single dose for optimum bacterial reduction in the GIT [29,104]. However, in-depth research is needed on the adverse impact of multiple doses, such as the production of phage-resistant mutants, host immune responses or feasibility in farm settings.

Furthermore, enteric capsules might be used for protecting phages from degradation in the GIT, as enteric capsules pass through the stomach intact and break down in the small intestine to release the phage. The capsules are coated with multiple layers of ethyl cellulose (water-impermeable polymer) and acid-resistant polymer (Eudragit S100), which make them resistant to the low acidic pH of the stomach or gizzard but sensitive to nearly neutral pH (5.5–7) of the small intestine, resulting in dissolution in the small intestine [108,109]. However, no in vivo studies have been conducted yet to determine the efficacy of enteric capsules for oral delivery of phage in food animals.

## 7. Research Gaps and Future Perspectives

Due to the consumption of animal-derived food products by humans, safety and efficacy are crucial for the development of effective phage-based therapeutics to control infectious diseases in food animals. Therefore, research needs to be conducted focusing on under-explored areas to support the broader application of phages and mitigate regulatory hurdles in animal production and health. Firstly, considerable variations in pH and thermal stability have been observed among phages. Therefore, research is needed to identify the molecular determinants of the stability differences that can help identify more robust phages or develop desirable phages through advanced molecular techniques such as genetic engineering. Secondly, the humoral immune response to orally administered phages remains poorly understood. Particularly, how phages interact with mucosal immune components and the mechanisms by which they may translocate from the gut into systemic circulation are not well defined. Detailed immunological and pharmacokinetic studies are necessary to enhance the safety and effectiveness of phage therapy in food-producing animals. Thirdly, most studies carried out to determine phages’ GI stability have their trials on laboratory animal models such as rats and mice. Only a few trials were conducted on food animals. As a result, the interactions between phages and the diverse physicochemical and biological components of the GIT remain poorly characterized. Therefore, more in vivo research needs to be conducted on food animals to determine phage stability in the GI environment. Fourthly, the methods used to assess phage stability in the GIT are not definitive. Hence, it is essential to establish standardized laboratory protocols for simulating GI conditions and efficiently assessing phage viability. In Fifth, although microencapsulation has emerged as a promising strategy to protect phages from degradation in the stomach, results across studies have been inconsistent. Further research should focus on the release rate of the encapsulated phages from the microparticles in the gut, optimization of the variability of phages, the extent of acid protection of the encapsulated phages, effective encapsulation techniques, and exploration of other strategies, including delivery through enteric capsules, to enhance phage survival and therapeutic efficacy following oral administration. Lastly, to promote the practical application of oral phage therapy, more attention needs to be paid to the production process under Good Manufacturing Practices (GMP), ensuring batch consistency, developing quality control criteria (such as identification, purity, and stability), improving storage methods, and formulating regulatory guidelines.

## 8. Conclusions

OPT offers a straightforward and non-invasive approach for managing gastrointestinal bacterial infections, especially AMR in food animals. However, low pH, digestive enzymes, bile salts, immune components, and ionic factors can significantly reduce phage viability and efficiency. In the laboratory, isolated phages are exposed to a range of pH, temperature, ions, and enzymes, and their titer is then determined, hence, titer variations are observed for determining stability. Microencapsulation, co-encapsulation, enteric capsules, pre-treatment with antacids or alkaline water, and repeated dosing have been explored to enhance phage survival and therapeutic outcomes. Despite these advancements, therapeutic outcomes in in vivo animals remain limited and inconsistent. Therefore, future research should aim to identify molecular determinants of phage stability under GI conditions, understand the role of gut-associated humoral immune responses, and validate phage stability in food animals through in vivo studies. Combined multifactorial stability testing rather than individual parameter testing could provide accurate and precise stability data, which enhances phage success rate at an oral route. Moreover, optimization of encapsulation techniques in collaboration with pharmacology and biotechnology and research on the estimation of the efficacy of novel approaches are essential for improving phage stability, storage, and efficacy in the oral route. Nevertheless, this review has several limitations, including the limited number of studies available in food-producing animals compared with laboratory animal models, which may introduce study bias. In addition, the risk of bias was not formally assessed, as no meta-analysis was performed, which may have contributed to selection bias. Finally, the review was not registered in PROSPERO, and a review protocol was not prepared in advance.

## Data Availability

All data are presented in this manuscript and additional data are provided in Supplementary Files.

## Supplementary Materials

The following supporting information can be downloaded at: https://www.mdpi.com/article/10.3390/v18050544/s1. Supplementary File: Supplementary Table S1. Heterogeneousness of experimental conditions and pH stability outcomes across included studies and List of research articles used for pH and thermal stability analysis [110,111].

## Abbreviations

The following abbreviations are used in this manuscript:

GIT	Gastrointestinal tract
OPT	Oral phage therapy
GDP	Gross domestic product
AMR	Antimicrobial resistance
SGF	Simulated gastric fluid
NaCl	Sodium chloride
CaCO_3_	Calcium carbonate
HCl	Hydrochloric acid
GALT	Gut-associated lymphoid tissue
Ig	Immunoglobulin
SM	Saline magnesium
LB	Luria-Bertani
PBS	Phosphate buffer saline
TSB	Tryptic soy broth
TM	Tris-Magnesium
NB	Nutrient broth
THB	Todd–Hewitt broth
ELISA	Enzyme-linked immunosorbent assay
DLA	Double-layer agar
IL	Interleukin
TNF	Tumor necrosis factor
TGF	Transforming growth factors
TLR	Toll-like receptor
