# Supplementary material for: Current Challenges and Potential Strategies to Enhance Efficacy of Oral Phage Therapy in Food Animals: A Systematic Review with Quantitative Analysis
Source: Viruses. 2026 May 8;18(5):544. doi: 10.3390/v18050544 (PMC13211687; doi:10.3390/v18050544)
Supplement: Supplementary file 1 [file viruses-18-00544-s001.zip › viruses-4281613-supplementary.pdf]

**Supplementary Table S1.** Heterogeneousness of experimental conditions and pH stability outcomes across included studies

| Phage name | Bacterial host | Animal model | Buffer system | pH exposure period (hours) | Temperature (˚C) | Titration method | Key findings | References |
|---|---|---|---|---|---|---|---|---|
| A221 | *E. coli* | Pigs | SM buffer | 2 | 37 | Double layer agar (DLA) | Stable: pH-5-9<br><br>Inactivated: 2-4 & 10-11 | Mao et al., 2023 |
| NJ12 and EP01 | *Salmonella* spp. and *E. coli* | Pigs | SM buffer | 2 | 37 | DLA | Stable: pH-6-11<br><br>Titer declined: pH-4-5 & 12<br><br>Inactivated: 1-3 & 13-14 | Li et al., 2024 |
| vB_EcoS_GN06 | *E. coli* | Chicken | TM buffer | 2 | 37 | DLA | Stable: pH-5-9<br><br>Titer declined: pH-4 & 10-11<br><br>Inactivated: pH-2-3 & 12 | Wang et al., 2022 |
| PEC9 | *E. coli* | Chicken | PBS | 1 | 37 | DLA | Stable: pH-3-11<br><br>Titer declined: pH-12<br>Inactivated: pH-2 & 13 | Yao et al., 2023 |
| *Escherichia* phage AG- MK-2022. Basu | *E. coli* | Chicken | SM buffer | 1 | 37 | DLA | Stable: pH-4-11<br><br>Titer declined: pH-3 & 12 | Karami et al., 2024 |

| | | | | | | | | |
|---|---|---|---|---|---|---|---|---|
| | | | | | | | Inactivated: pH-1-2 & 13-14 | |
| vB_EcoM_CE1 | *E. coli* | Chicken | LB broth | 3 | 37 | DLA | Stable: pH-4-10<br><br>Inactivated: pH-2-3 & 11-13 | Tang et al., 2023 |
| vB_SalP_LDW16 | *Salmonella* spp. | Chicken | SM buffer | 1 | 37 | DLA | Stable: pH-6-12<br><br>Titer declined: pH-3-5 & 13<br><br>Inactivated: pH-1-2 | Cao et al., 2022 |
| EcSw | *E. coli* | Pigs | SM buffer | 1 | 37 | DLA | Stable: pH-5, 7 & 9<br><br>Titer declined: pH-2 & 11<br><br>Inactivated: pH-1 & 13 | Easwaran et al., 2015 |
| vB_EcoM-P896 | *E. coli* | Ducks | PBS | 1 | 25 | DLA | Stable: pH-3-10<br><br>Titer declined: pH-11<br><br>Inactivated: pH-1-2 & 12 | Zhang et al., 2024 |
| SMP | *Streptoccus suis* | Pigs | Todd-Hewitt broth | 1 | 37 | DLA | Stable: pH-5-9<br><br>Titer declined: pH-4 & 10-11 | Ma and Lu, 2008 |
| Psq-1 | *E. coli* | Pigs | LB broth | 2 | 37 | DLA | Stable: pH-4-13<br><br>Inactivated: pH-1-3 & 14 | Wan et al., 2025 |

| | | | | | | | | |
|---|---|---|---|---|---|---|---|---|
| SEpBS-1 | *Salmonella* spp. | Chicken | Nutrient broth | 0.5 | 41 | DLA | Stable: pH-5 & 7<br><br>Titer declined: pH-3 & 9 | Wanasawaeng et al., 2025 |
| *Escherichia* coli O157:H7 phage | *E. coli* | Cattle | Tryptic soy broth | 24 | 37 | DLA | Stable: pH-5, 7 & 9<br><br>Titer declined: pH-1-2 & 11 | Litt and Jaroni, 2017 |
| LMP3 | *Listeria monocytogenes* | Cattle | Physiological saline | 1 | 37 | DLA | Stable: pH-4&7-9<br><br>Titer declined: pH-2-3 & 10-12 | Elsayed et al., 2023 |
| MSA6 | *Staphylococcus aureus* | Cattle | TM buffer | 1 | 37 | DLA | Stable: pH-4-10<br><br>Inactivated: pH-3 & 11 | Kwiatek et al., 2012 |
| SPW | *Staphylococcus aureus* | Cattle | LB broth | 3 | 37 | DLA | Stable: pH-4-9<br><br>Inactivated: pH-2-3 & 10 | Li and Zhang, 2014 |
| *Salmonella* Typhimurium phages | *Salmonella* spp. | Pigs | SM buffer | 2 | 37 | DLA | Stable: pH-3-4<br><br>Titer declined: pH-2 | Albino et al., 2014 |
| SPFM (2, 4, 10, 14, 17, 19) | *Salmonella spp.* | Pigs | SM buffer | 1 | 25 | Plaque assay | Stable: pH-4-12<br><br>Titer declined: pH-3<br><br>Inactivated: pH-1-2 & 13-14 | Thanki et al., 2022 |
| Bonnie and Clyde | *Streptoccus suis* | Pigs | SM buffer | 24 | 37 | DLA | Stable: pH-4-10<br><br>Titer declined: | Osei et al., 2025 |

| | | | | | | | | |
|---|---|---|---|---|---|---|---|---|
| | | | | | | | pH-3 & 11-12<br><br>Inactivated: pH-2 & 13 | |
| vB_EcoP_SD2 | *E. coli* | Pigs | LB broth | 12 | 37 | DLA | Stable: pH-4-11<br><br>Inactivated: pH-1-3 & 12 | Ren et al., 2025 |
| PY223 | *Bordetella bronchiseptica* | Pigs | SM buffer | 1 | 37 | DLA | Stable: pH-5, 7 & 9<br><br>Titer declined: pH-3<br><br>Inactivated: pH-11 | Huang et al., 2025 |
| XAM237 | *E. coli* | Pigs | LB broth | 1 | 37 | DLA | Stable: pH-5-10<br><br>Titer declined: pH-2-4<br><br>Inactivated: pH-1 & 11-12 | Chai et al., 2025 |
| vB_CpeP_15N3 | *Clostridium perfringens* | Pigs | Tryptic soy broth | 1 | 37 | DLA | Stable: pH-5-10<br><br>Titer declined: pH-3-4 & 11-12<br><br>Inactivated: pH-2 | Wu et al., 2025 |
| fmb-p1 | *Salmonella* spp. | Ducks | LB broth | 1 | 37 | DLA | Stable: pH-4-10<br><br>Titer declined: pH-2-3 & 11-13 | Wang et al., 2017 |
| vECPW8 | *E. coli* | Chicken | SM buffer | 1 | 37 | DLA | Stable: pH-4-10<br><br>Titer declined: pH-3 & 11-12 | Wintachai et al., 2024 |

| | |
|---|---|
| | Inactivated: pH-1-2 & 13-14 |

**List of research articles used for pH and thermal stability analysis**


1. Mao, X., Wu, Y., Ma, R., Li, L., Wang, L., Tan, Y., Li, Z., Liu, H., Han, K., Cao, Y. and Li, Y., 2023. Oral phage therapy with microencapsulated phage A221 against *Escherichia coli* infections in weaned piglets. BMC Veterinary Research, 19(1), p.165.
2. Li, L., Han, K., Mao, X., Wang, L., Cao, Y., Li, Z., Wu, Y., Tan, Y., Shi, Y., Zhang, L. and Liu, H., 2024. Oral phages prophylaxis against mixed *Escherichia coli* O157: H7 and *Salmonella* Typhimurium infections in weaned piglets. Veterinary Microbiology, 288, p.109923.
3. Wang, L., Tan, Y., Liao, Y., Li, L., Han, K., Bai, H., Cao, Y., Li, J., Gong, Y., Wang, X. and Peng, H., 2022. Isolation, characterization and whole genome analysis of an avian pathogenic *Escherichia coli* phage vB_EcoS_GN06. Veterinary Sciences, 9(12), p.675.
4. Yao, L., Bao, Y., Hu, J., Zhang, B., Wang, Z., Wang, X., Guo, W., Wang, D., Qi, J., Tian, M. and Bao, Y., 2023. A lytic phage to control multidrug-resistant avian pathogenic *Escherichia coli* (APEC) infection. Frontiers in Cellular and Infection Microbiology, 13, p.1253815.
5. Karami, M., Goudarztalejerdi, A., Mohammadzadeh, A. and Berizi, E., 2024. In vitro evaluation of two novel *Escherichia* bacteriophages against multiple drug resistant avian pathogenic Escherichia coli. BMC Infectious Diseases, 24(1), p.497.
6. Manohar, P., Tamhankar, A.J., Lundborg, C.S. and Ramesh, N., 2018. Isolation, characterization and in vivo efficacy of *Escherichia* phage myPSH1131. PloS one, 13(10), p.e0206278.
7. Tang, Z., Tang, N., Wang, X., Ren, H., Zhang, C., Zou, L., Han, L., Guo, L. and Liu, W., 2023. Characterization of a lytic *Escherichia coli* phage CE1 and its potential use in therapy against avian pathogenic Escherichia coli infections. Frontiers in Microbiology, 14, p.1091442.
8. Cao, S., Yang, W., Zhu, X., Liu, C., Lu, J., Si, Z., Pei, L., Zhang, L., Hu, W., Li, Y. and Wang, Z., 2022. Isolation and identification of the broad-spectrum high-efficiency phage vB_SalP_LDW16 and its therapeutic application in chickens. BMC veterinary research, 18(1), p.386.
9. Easwaran, M., Paudel, S., De Zoysa, M. and Shin, H.J., 2015. Functional characterization of a novel lytic phage EcSw isolated from *Sus scrofa domesticus* and its potential for phage therapy. Molecular and Cellular Probes, 29(3), pp.151-157.

10. Zhang, H., Su, X., Zheng, X., Liu, M., Zhao, C., Liu, X., Ma, Z., Zhang, S. and Zhang, W., 2024. vB_EcoM-P896 coliphage isolated from duck sewage can lyse both intestinal pathogenic *Escherichia coli* and extraintestinal pathogenic *E. coli*. International Microbiology, pp.1-12.
11. Ma, Y.L. and Lu, C.P., 2008. Isolation and identification of a bacteriophage capable of infecting *Streptococcus suis* type 2 strains. Veterinary microbiology, 132(3-4), pp.340-347.
12. Wan, S., Li, N., Habib, S., Zheng, P., Li, Y., Liang, Y. and Qu, Y., 2025. Biological Characteristics and Whole-Genome Analysis of a Porcine *E. coli* Phage. Veterinary Sciences, 12(1), p.57.
13. Wanasawaeng, W., Thomrongsuwannakij, T. and Chansiripornchai, N., 2025. Isolation, Characterization, and Application of Bacteriophage for *Salmonella* Control in Broiler Chickens. Veterinary Medicine International, 2025(1), p.6502225.
14. Litt, P.K. and Jaroni, D., 2017. Isolation and Physiomorphological Characterization of *Escherichia coli* O157: H7-Infecting Bacteriophages Recovered from Beef Cattle Operations. International journal of microbiology, 2017(1), p.7013236.
15. Elsayed, M.M., Elkenany, R.M., Zakari, A.I. and Badawy, B.M., 2023. Isolation and characterization of bacteriophages for combating multidrug-resistant Listeria monocytogenes from dairy cattle farms in conjugation with silver nanoparticles. BMC microbiology, 23(1), p.146.
16. Kwiatek, M., Parasion, S., Mizak, L., Gryko, R., Bartoszcze, M. and Kocik, J., 2012. Characterization of a bacteriophage, isolated from a cow with mastitis, that is lytic against *Staphylococcus aureus* strains. Archives of virology, 157, pp.225-234.
17. Li, L. and Zhang, Z., 2014. Isolation and characterization of a virulent bacteriophage SPW specific for *Staphylococcus aureus* isolated from bovine mastitis of lactating dairy cattle. Molecular biology reports, 41, pp.5829-5838.
18. Thanki, A.M., Clavijo, V., Healy, K., Wilkinson, R.C., Sicheritz-Pontén, T., Millard, A.D. and Clokie, M.R., 2022. Development of a phage cocktail to target *Salmonella* strains associated with swine. Pharmaceuticals, 15(1), p.58.
19. Osei, E.K., O'hea, R., Cambillau, C., Athalye, A., Hille, F., Franz, C.M., O'doherty, Á., Wilson, M., Murray, G.G., Weinert, L.A. and Manzanilla, E.G., 2025. Isolation of phages infecting the zoonotic pathogen Streptococcus suis reveals novel structural and genomic characteristics. Microbiological Research, 296, p.128147.
20. Ren, Q., Wang, Z., Ge, Y., Huang, Y., Zhang, W., Liu, C., Li, Y. and Cao, S., 2025. Biological characterization of novel *Escherichia coli* O157: H7 phages and their bacteriostatic effects in milk and pork. Frontiers in Microbiology, 16, p.1516223.

21. Huang, X., Hou, Y., Zhao, M., Chen, J., Zhu, Z., Liu, H., Wang, M., Hua, L., Chen, H., Wu, B. and Peng, Z., 2025. Identification of the broad-spectrum lytic *Bordetella* phage and assessments of its potential for combating Bordetella infections. Virology, p.110545.
22. Chai, J., Sun, H., Schwarz, S., Huang, Y., Xie, S., Xu, Q., Lin, L., Ma, C., Hou, J., Zhu, Y. and Zhang, W., 2025. Isolation, characterization, and application of the novel polyvalent bacteriophage vB_EcoM_XAM237 against pathogenic *Escherichia coli*. Veterinary Research, 56(1), p.90.
23. Wu, Y., Zhang, L., Zheng, H., Huang, W., Zhang, X., Ji, W., Ma, R., Mao, X., Huang, Y., Liu, X. and Zeng, J., 2025. Oral bacteriophage therapy effectively prevent and control of *Clostridium perfringens* type c infections in newborn piglets. Veterinary Microbiology, 300, p.110330.
24. Wang, C., Chen, Q., Zhang, C., Yang, J., Lu, Z., Lu, F. and Bie, X., 2017. Characterization of a broad host-spectrum virulent *Salmonella* bacteriophage fmb-p1 and its application on duck meat. Virus Research, 236, pp.14-23.
25. Wintachai, P., Thaion, F., Clokie, M.R. and Thomrongsuwannakij, T., 2024. Isolation and Characterization of a Novel *Escherichia* Bacteriophage with Potential to Control Multidrug-Resistant Avian Pathogenic *Escherichia coli* and Biofilms. Antibiotics, 13(11), p.1083.
